# Drug Vaping: From the Dangers of Misuse to New Therapeutic Devices

**DOI:** 10.3390/toxics4040029

**Published:** 2016-12-16

**Authors:** V. Varlet

**Affiliations:** Forensic Toxicology and Chemistry Unit, University Centre of Legal Medicine Lausanne—Geneva, Lausanne CH-1011, Switzerland; Vincent.varlet@chuv.ch

**Keywords:** drug vaping, e-cigarette, e-liquid, vaping alternative use, vaping misuse

## Abstract

Users of e-cigarettes are unwitting volunteers participating in a worldwide epidemiological study. Because of the obvious benefits of e-cigarettes compared with traditional cigarette smoking, these electronic devices have been introduced all around the world to support tobacco smoking cessation. Same potential harm reduction could be considered by cannabis vaping for marijuana smokers. However, the toxicities of liquids and aerosols remain under investigation because although the use of e-cigarettes is likely to be less harmful than traditional cigarette smoking, trace levels of contaminants have been identified. Simultaneously, other electronic devices, such as e-vaporisers, e-hookahs or e-pipes, have been developed and commercialised. Consequently, misuse of electronic devices has increased, and experimentation has been documented on Internet web fora. Although legal and illegal drugs are currently consumed with these e-devices, no scientific papers are available to support the observations reported by numerous media and web fora. Moreover, building on illegal drug vaping and vaporisation with e-devices (vaping misuse), legal drug vaping (an alternative use of vaping) could present therapeutic benefits, as occurs with medical cannabis vaporisation with table vaporisers. This review seeks to synthesise the problems of e-cigarette and liquid refill toxicity in order to introduce the dangers of illegal and legal drugs consumed using vaping and vaporisation for recreational purposes, and finally, to present the potential therapeutic benefits of vaping as a new administration route for legal drugs.

## 1. Introduction

Whether tobacco smoking cessation with e-cigarettes is safe is currently quite a controversial topic. Some studies have noted high amounts of toxicants in the liquid refills [1]. Other studies focused on the contaminants in the vapours, primarily formaldehyde [2]. However, these latter studies were criticised by other scientists because the necessarily high e-cigarette settings led to “dry-puffs” with aversive organoleptic properties resulting in inaccurate vaping conditions [3]. Consequently, the analogy to toasters developed by Hajek is sensible: current commercialised toasters have settings that allow burnt toast, which contain carcinogenic substances. Individuals who like burnt toast are possibly aware of the carcinogenic burden being consumed [4]. Therefore, unless the toaster industry is obliged to manufacture toasters with restricted settings that avoid overheating, there is no reason to commercialise standardised e-cigarettes to avoid dry puffs. Moreover, it could be a moot point, considering the new generation of modified e-cigarettes such as modified powerful e-cigarettes (MODs) and do-it-yourself (DIY) devices. It appears more important to communicate guidelines for users and to make vapers aware of the uncontrolled generation of hazardous substances produced at high settings. Even if electronic devices such as e-cigarettes can generate contaminants in the aerosols vaped, these contaminants will likely continue to be generated at lower levels than those identified in the smoke of traditional cigarettes [5].

On the edge of this debate, other questions surrounding e-cigarettes are emerging, legitimized by vapers and illegal drug consumers’ experiments documented primarily on the Internet and web fora. For example, e-cigarettes can be misused for consumption of illegal drugs such as cannabinoids (cannavaping), which can be dissolved in liquid refills [6,7]. Other devices such as e-vaporisers are specifically designed for smoking or/and dabbing, i.e., the direct consumption of cannabis in concentrated (semi)solid forms [8,9]. Other illegal substances can be consumed with these new e-cigarette techniques. Amphetamines, cocaine and heroin derivatives that can be smoked may also be easily and discretely vaped. Some of these drugs, such as cannabinoids, are already consumed via medical vaporisers in therapeutic applications whereas others are illegally consumed and inhaled by glass pipe vaporisation for recreational use. The development of e-devices should therefore allow legal and illegal drug consumption via pen-vaporisers and e-cigarettes.

However, if these new developments can lead to misuse, such developments may also offer a new administration route for legal drugs. Vaping may be more efficient than mouth spraying and the oral route. Indeed, ingested drugs require higher doses to overcome gastrointestinal absorption limitations and first-pass metabolism through the liver before reaching the bloodstream, whereas inhaled compounds have direct access to the bloodstream without being metabolised. Therefore, a prospective therapeutic application of e-cigarette use could be as a new drug form for controlled self-administration.

The purpose of this review is to present the current alternative uses and misuses of e-cigarettes and pen-vaporisers in vaping legal and illegal drugs and to open the discussion of the possibility of using standardised e-cigarettes and controlled liquid refills fortified with legal drugs as new galenic formulations using a new therapeutic administration method.

## 2. Electronic-Device-Related Toxicities

Electronic cigarettes are hand-held devices mimicking traditional cigarettes by the battery-powered atomisation of a flavoured (or unflavoured) liquid refill-containing (or not-containing) nicotine. E-cigarettes represent a sector of the electronic nicotine delivery systems (ENDS). According to the settings of the device, different temperatures can be achieved, producing mists/aerosols, or vapours. High temperatures may be sufficient to transform the liquid refill into vapours, defined as the gaseous state of a substance that is normally solid or liquid, in the close vicinity of the coil. Therefore, because vapours can be generated, the act of “smoking” e-cigarettes is called “vaping”, and the users of e-cigarettes, “vapers”. However, according to the geometry of the device and because the vapour must be “vapable” (i.e., at an aerosol temperature reaching the mouth below 60 °C), the temperature of the generated vapour decreases, resulting in a product that appears more like a mist or an aerosol, defined as a liquid finely suspended in air (for mist) or a gas/gas mixture (for aerosol). Because no combustion occurs, the burden of contaminants identified in cigarette smoke and present in the aerosol is considerably lower than in the smoke of traditional cigarettes, even if more research should be performed with respect to this point to cover all contaminants present in the aerosol of e-cigarettes.

However, several concerns regarding the innocuity of these devices have arisen, primarily focused on the toxicity of liquid refills and of generated aerosols from these liquids. Considering the potentially huge benefits of e-devices in tobacco smoking cessation, many scientists have studied these liquids and aerosols.

### 2.1. Toxicity of Liquid Refills

Commercialised liquid refills, or e-liquids, comprise edible solvents (primarily propylene glycol, PG, and vegetal glycerine) in varying proportions. Different nicotine concentrations can be used to allow users (non-smokers and smokers alike) to modify their nicotine exposure to fit their desires and needs. Many flavours are available to the consumer, and aromatic compounds can therefore be identified in the e-liquid. Similarly, some e-liquids contain a small amount of ethanol, known to improve the pleasant “throat hit”; however, the ethanol presence can also come from impurities in flavourings during their manufacture as aromatic support as well as in PG or glycerine. Finally, it is also possible to identify traces of water although its presence is generally not required to ensure the best stability. All of these allegedly food-grade components can also be purchased individually in specialised shops or on the Internet to allow the consumer to prepare his own homemade e-liquid by himself. In this case, the individual molecules added by the user according to their own recipe could raise some concerns. Indeed, even food-grade components could become toxic because of the doses ingested through lungs. Moreover, food-grade components and chemicals used in e-liquids have been approved for gastrointestinal ingestion, but not for inhalation. 

Toxicity concerns regarding liquid refills were observed after several poisonings of vapers. Pathologies suffered by vapers were reported to be primarily pneumonia and lipoid pneumopathy [10]. Pneumonia is thought to be particularly associated with heavy vaping. It was shown that e-cigarettes are not be safer than tobacco cigarettes when effects related to exhaled nitric oxide (eNO) reduction are considered [11,12]; however, these results depend on the e-device model, vaping conditions, etc. Heavy use can negatively affect pulmonary function, which is why manufacturers recommend stopping for 30 min after taking 16 puffs to avoid complications, as reported in already published work [13]. This result is thought to be primarily related to solvents and atomised compounds rather than nicotine. Some studies reported that repeated inhalation of vapours containing PG was associated with acute cough and dry throat and decrease in lung function [12]; and glycerine, even if generally considered to be non-hazardous and with low oral toxicity, may be linked to lipoid pneumonia, which is associated with chronic use [14]. Some of these cases may be attributed to insufficient cleaning of the device. 

In addition, concerns regarding liquid composition itself have been reported. Accidental ingestion by infants [15] and children [16,17] or deliberate ingestion [18] and injection [19] for suicidal purposes by adults have led to several poisonings of different magnitudes. Nicotine appears to be the toxic agent in those cases.

All of these results and observations have led scientists to analyse the liquid compositions. In addition to nicotine, which can be intentionally mixed into the e-liquid, other toxic substances have been identified. The majority of these substances are likely manufacturer contaminants introduced by the initial, insufficiently pure compounds or unexpected reactions between components during formulations.

Tobacco nitrosamines such as impurities in nicotine were reported in several analyses [20,21,22,23,24]. These compounds are known to be carcinogenic in vivo in animals but there is still no evidence that tobacco nitrosamines are carcinogenic for humans. Terpenic compounds, often added to confer a fruity taste to the e-liquid, have been measured at problematic levels [1]. Chemicals such as glycols [1,23], diacetyl, acetyl propionyl, cumarine and acetamide have been detected [25,26] as well as pharmaceutical agents such as aminotadalafil and rimonabant [27]. Cytotoxicity in e-liquid was also positively linked to flavouring contents using embryonic and adult models, showing that the quality of the product is important when choosing an e-liquid [28].

Therefore, according to the quality of the liquid refill, identifiable contaminants are present in varying amounts. These amounts are rarely of toxic concern if vaped; however, considering the high variability among the marketed products (particularly on the Internet), controls and laws regarding e-cigarette liquid refills should be rapidly enacted to avoid the commercialisation of impure and potentially unsafe e-liquids [29]. Similarly, special packaging with safety lids should be developed to avoid ingestion by children.

### 2.2. Toxicity of Aerosols

Although specific cases have highlighted the presence of contaminants, the majority of liquid refills may be safe for short-term use. Only chronic heavy use and vaping misuse (modifications of the e-device, homemade recipes, ingestion/injection, etc.) lead to problematic concerns, even if chronic light use and chronic normal use have not been shown to be safe. However, generating vaping contaminants in the aerosol remains possible even using safe liquids. Indeed, the e-device settings such as battery power, design of clearomisers/cartomisers, puff duration and frequency can promote adverse reactions among the e-liquid components.

Numerous studies have investigated the toxicity of the aerosols generated by e-cigarettes. The aerosol cytotoxicity [30,31,32]; the amounts of carbonyls [2,5,33,34,35,36], diethylene glycol [37] and glycols [35]; volatile compounds and solvents [5,35,38], nitrosamines [5,35,36]; metals [5,39] and flavourings [40]; and the size of particulates [41,42,43,44,45] have been studied over the last five years.

The results appear to be random and subjectively interpreted. Indeed, each quantification should consider the vaping design and conditions. Moreover, differences in materials and methods, liquid composition, vaping conditions, the position of the temperature probe to measure the atomisation temperature, and e-device designs render objective interpretations difficult [46,47,48,49].

Nevertheless, use of high settings of e-cigarettes is known to promote contaminants. Overheating of glycerine and PG have been reported to generate carbonyl contaminants (such as acrolein, formaldehyde, acetaldehyde, [E]-2-alkenals, alkanals, and tolualdehydes) [29,35,39]. Overheating of the device could also create metallic [50] and polycyclic contaminants [35,38,44].

However, the use of e-cigarette settings leading to high burdens of these contaminants also generates unpleasant “dry puffs”. These repellent “dry puffs” should cause sufficient organoleptic flaws to lead to avoidance in vapers [3]. However, some vapers may like “burnt” flavours and be subjected to these toxic compounds. Even if this is dangerous from a health point of view, in other contexts consumers are exposed to the possibility of contamination via consumption of polycyclic aromatic hydrocarbons [51] and heterocyclic amines [52] in roasted/smoked food. Moreover, more research should be led on this topic in order to link objectively higher levels of contaminants with significant burnt taste due to high temperature settings.

Thus, as the presence of contaminants in the aerosols has been demonstrated, some studies have concluded that these contaminants could occur in amounts lower than in conventional tobacco cigarette smoke [53,54]. Thus, the benefits of vaping compared with tobacco smoke could be promoted among individuals who want to quit smoking. This promotion should target specific groups of individuals to avoid public advertisement, which may seduce young people attracted by the technological features of e-devices. However, these recommendations remain controversial because of the lack of long-term scientific data.

### 2.3. Solutions

The only solution to contaminant generation is standardisation of safe e-materials. However, this solution is illusory because all of the ingredients are legally sold and procedures to prepare homemade liquid refills are easily available. All of the ingredients are legal and edible, from solvents to flavourings. Standardisation of commercialised liquids will solve the majority of the problem (in terms of endogenous toxic compounds such as tobacco alkaloids) but will not eradicate contaminant generation because of the “do-it-yourself” possibility of homemade products. Innovation, safety and quality standards are being introduced to minimize risks.

Limiting users’ access to e-cigarette settings could be a solution, although persons with electronic skills can easily upgrade their e-cigarettes and transform them into modified powerful e-cigarettes (MODs). Procedures are also easily available on the Internet. If official vaping championships are organised by e-cigarette manufacturers, other unofficial “power vaping” championships are also organised, for example “power vaping”. This refers to the generation of the widest and longest “cloud” exhaled from vaper’s mouth and is obtained primarily with MODs. Therefore, although the standardisation of commercialised electronic devices will solve much of the problem, standardisation will not eradicate power vaping because of the “do-it-yourself” possibility of homemade devices, strongly dependent on the electronic skills of users. Correct user information and communication should have been given to guide him/her in avoiding risky behaviours.

Because toxicities in liquid refills and aerosols cannot be completely regulated and avoided, it is more important to communicate guidelines for users and make vapers aware of the uncontrolled generation of hazardous substances produced at high settings than to promote standardisation of liquids and devices.

## 3. Illegal Drug Consumption with Electronic Devices

### 3.1. Illegal Drug Vaping

Illegal drug vaping involves the use of conventional e-cigarettes and adapted liquid refills. The drug must be dissolved in the liquid, which can either be a commercialised or a homemade liquid.

The primary problem with this type of misuse is solubilisation of the drug in the liquid component. Commercialised liquid refills comprise various amounts of edible solvents (primarily PG and vegetal glycerine), trace amounts of ethanol (as eventual flavouring support) and water. Illegal drug vapers should therefore know the solubility of the illegal substance in the proposed liquid refill. Moreover, the viscosity of the vapable solvents and the hydrophobicity of many illegal substances may be a reason not to prepare such vapable recreative liquids. Nevertheless, all drugs that can be dissolved in a liquid refill can theoretically be vaped [9].

#### 3.1.1. Cannavaping 

Among the illegal drugs susceptible to vaping, cannabis is the most frequently cited on web fora [55,56]. The vaping of cannabis dissolved in a liquid refill has been named “cannavaping”. This is the only illegal substance (except in certain countries in which cannabis is legal) whose vaping has been studied objectively [7]. The preparation of cannabinoids-enriched vapable liquid refills was determined to be problematic because of the form of the cannabis to be dissolved (solid, paste, heads, etc.) and the physicochemical properties of tetrahydrocannabinol (THC, the active substance of cannabis), which is not easily soluble in viscous and hydrophilic solvents. 

**Figure 1:**
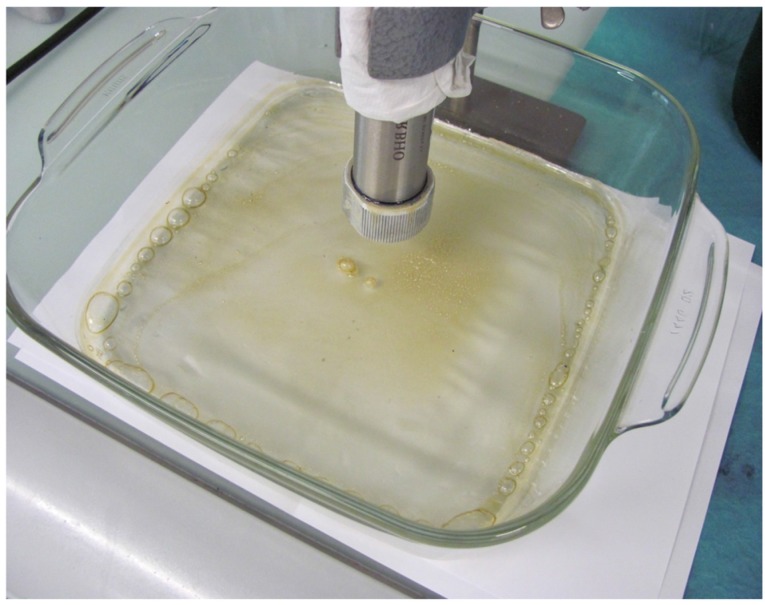
Butane Hash Oil (BHO) homemade extraction.

In fact, recipes on the Internet present protocols for cannabis bud/leaf solvent extraction (maceration, infusion or percolation) with various solvents (alcoholic or non-alcoholic) to maximise the extraction recovery yield and the production of tinctures that can be further concentrated by solvent evaporation. Butane cannabinoid extraction is one of the most efficient procedures, leading to butane honey oil, or butane hash oil (BHO), a waxy, sticky and highly concentrated form of cannabinoids (Figure 1) [57,58]. Further filtrations and other winterisation steps (elimination of particulates and triglycerides) can eventually be conducted. The resulting BHO comprises 70%–75% of inactive THC-A, a precursor of THC. THC-A must be decarboxylated into THC to become psychoactive. This decarboxylation is generally achieved during the pyrolysis of cannabis buds in cigarettes while smoking. Based on e-cigarette and puff conditions, the temperature reached during liquid vaporisation is not sufficiently high and maintained to generate satisfactory THC recovery yields in the aerosol. Therefore, a preliminary decarboxylation is mandatory. However, this step is quite difficult from an organoleptic perspective because the step requires temperatures above 100 °C for several hours. At such temperatures, the compromise between efficient decarboxylation of THC-A into THC, limiting the loss of the natural terpenoids responsible for the organoleptic qualities desired by consumers, and limiting the generation of thermally neoformed compounds and contaminants, is quite challenging. Consequently, homemade preparations of cannabis forms ready to be solubilised in commercialised or homemade liquid refills appear to be quite complex.

Another conclusion of this study is that pure PG is a better candidate for BHO solubilisation and that important percentages of BHO should be dissolved in liquid refills in order to feel the first psychoactive effects. A minimum of 200 puffs (commercial liquid refill) or 95 puffs (pure PG), both spiked with decarboxylated BHO (10% *w*/*w*), could be sufficient to reach minimal therapeutic or recreational effects (equivalent to 1.5 mg of THC, i.v.). However, when avoiding glycerine in the solvent, a decrease of the organoleptic qualities is expected because glycerine is responsible for the “white vapour” and its presence is wished by conventional vapers. Moreover, increasing the BHO percentage in the liquid refill leads to an even more difficult solubilisation. 

Consequently, although illegal cannavaping is theoretically possible, this misuse would probably undergo marginal development. The poor solubility of BHO in commercial liquid refills (particularly refills with a high glycerine content) prevents high BHO concentrations, which are desired by illegal cannavapers to feel rapidly psychoactive effects. However, the wide diversity of e-devices could lead to other types of cannavaping, which in turn, could lead to totally different results.

#### 3.1.2. Synthetic Cannavaping

Although web fora provide extensive protocols to prepare homemade cannabinoid-enriched liquid refills, such refills are not in fact commercialised on the Internet. However, other illegal cannabinoid-vapable liquids contain synthetic cannabinoids are marketed [59].

Synthetic cannabinoids are a component of new psychoactive substances (NPS). These synthetics represent a relatively new class of designer drugs that have recently emerged as popular alternatives to marijuana and are referred to as “legal highs” or “Spice”. These drugs are available on the Internet and sold in many head shops under the guise of innocuous products such as herbal blends, incense, potpourri, aromatherapy, e-cigarette liquid refills or air fresheners [60]. Spice contains synthetic cannabinoids that bind to cannabinoid-like receptors. Synthetic cannabinoids are stronger than natural cannabis [61] and have led to fatal intoxications [62]. Many of them were developed in the 1990s at Clemson University and are named in series. The JWH is a series named after investigator John W. Huffman; the HU series was developed at Hebrew University by Raphael Mechoulam; the (Charles Pfizer) CP series was developed by Charles Pfizer; and the AM series was developed by Alexandros Makriyannis. The most common cannabinoids in the US belong to the JWH series, namely JWH-018. Unlike opioid drugs such as heroin and morphine, synthetic cannabinoids have no available antidote. Synthetic cannabinoids are full agonists at the cannabinoids (CB) receptors and, unlike regular cannabis, do not contain cannabidiol, which has anxiolytic and antipsychotic properties. Chronic use of these drugs can lead to addiction syndrome and withdrawal symptoms similar to those observed in cannabis abuse [63]. Several European countries and, more recently, the United States (US), have placed restrictions on or banned the use and sale of these drugs because of their dangerous effects and abuse potential [64]. 

Synthetic cannabinoids are sold in various forms (crystalline, herbal, liquid, etc.). The solid form can be vaped by an e-cigarette but dissolves poorly in the vaping liquid, causing the drug to begin to crystallise over time [65]. However, some forms of these synthetic cannabinoids are available in liquid form and are easily solubilised in commercialised or/and homemade liquid refills. XLR-11, AB-PINACA, AB-Fubinaca and derivatives are the most active substances in liquid synthetic cannabinoids [66,67]. AB-PINACA and AB-Fubinaca, sold under the street name “Cloud 9” and also known as Hookah Relax, are both derivatives of JWH-018. These liquid synthetic cannabinoids have the street names Bizarro, Crown, Shisha, Mad Hatter, Bomb Marley, WTF, Diablo, Sexy Monkey, and others [68,69].

#### 3.1.3. Other Illegal Drug Vaping

Synthetic cannabinoids are most representative of liquid NPS. However, other illegal new liquid drugs are available, such as solid/crystalline NPS, which can be dissolved in liquid refills by the consumer or directly marketed in liquid form. Negative experiences were reported on the Internet concerning the liquid form of α-pyrrolidinovalerophenone (α-PVP or Flakka) [70]; α-PVP is a synthetic cathinone that acts as a stimulant and is known to cause excited delirium. Like numerous designer drugs (cathinones, cannabinoids, tryptamines, etc.) pharmacodynamics and pharmakinetics are generally unknown. Synthetic cathinones and tryptamines can cause overdoses more easily than synthetic cannabinoids because they are more potent [71]. However, for stability and transport reasons, these molecules are packaged and available in powder or crystalline forms. Because these synthetics can be ingested, snorted, smoked or injected, the solubilisation in liquid refill for vaping does not appear to be an issue. Moreover, although a small quantity is sufficient to feel the first psychoactive effects, vaping may be deemed inefficient by users who want to feel rapid psychoactive effects.

Another liquid drug concerns Gamma-Hydroxybutyrate (GHB), also known as the rape drug. GHB is a powerful synthetic drug that has euphoric and sedative effects. GHB is rapidly metabolised by the body, and the effects of the drug can be felt within fifteen to twenty minutes after ingestion. However, higher concentrations of GHB can cause dizziness, nausea, vomiting, confusion, seizures, respiratory depression, and intense drowsiness, sometimes leading to unconsciousness or coma and death [72]. Because strong effects can be achieved with extremely small amounts (e.g., a few drops, a capful), it is easy to overdose, particularly when the drug is mixed with alcohol or other drugs. GHB is the active molecule of Xyrem^®^, a legal drug available for the treatment of narcolepsy; however, the majority of the GHB being used today is of the “homemade” variety. Homemade GHB is made by non-professionals in their own “street labs”, kitchens, or bathtubs by mixing various chemical ingredients. Significant differences in the purity, concentration, and potency of various batches may occur. Recreational vaping of illegal GHB is theoretically possible although few comments have been posted on the Internet. 

Experiments concerning ethanol vaping have also been reported. People frequently add a few drops of alcohol to increase the throat hit; however, a quantity of approximately 2% is sufficient and avoids alcoholic effects. Some liquid refills already contain ethanol for this purpose or because the ethanol was used as flavouring support during the preparation of the liquid. The boiling point of ethanol is close to 78 °C. Consequently, in the majority of vaping settings, higher percentages of ethanol lead to ethanol aerosols, which can irritate the throat as reported several times by vapers on web fora. 

Other liquid refill preparations are also available on the Internet under the name e-juices. E-liquids containing plant extracts can be designed by this term, but it is not exclusively dedicated to these types of e-liquids. The packaging displays (natural) plant extracts and claims stimulant properties due to guarana content (*Paullinia cupana*; active compounds: xanthines such as caffeine and theobromine); other preparations are used as cannabis substitutes by recreational users as an alternative to illegal psychoactive plants. Indeed, the majority of these plants are not currently scheduled under federal law in the United States, including *Leonotis leonorus* (also known as wild dagga; active compound: leonurine, with similar effects to THC when smoked), and other Lamiales such as coleus (*Coleus blumei*, also known as *Plectranthus scutellarioides*; active compounds not identified, with psychedelic effects when chewed or smoked); Indian warrior (*Pedicularis densiflora;* active compounds not identified, with relaxing effects in tea blends); Kanna (*Sceletium tortuosum*; active compounds: mesembrine and tortuosine, with entheogenic and sedative effects when chewed, ingested, snuffed or smoked); Syrian Rue/harmal, (*Peganum Harmala*; active compounds: harmaline, harmine and harmol, with relaxing and emmenagogue effects when ingested); and wild lettuce or (*Lactuca virosa*; active compounds: *N*-methyl-β-phenethylamine, lactucin and lactucopicrin, with mild hypnotic and sedative effects when ingested or smoked). However, these liquid refills can be mixed with synthetic cannabinoids.

Plant extracts lead to problematic challenges because of the heterogeneity of national regulations. For example, *Salvia divinorum* (active compound: salvinorine) is forbidden in Australia, certain European countries and certain states in the US whereas other countries have only forbidden the consumption of this plant. 

Therefore, plant extracts can theoretically be vaped if dissolved in a liquid refill. However, such extracts are primarily available in powder form for transport and stability reasons although liquid forms can be purchased. The difficulty of dissolving these powders in viscous liquid refills renders them inadequate for vaping. However, the dissolution of plant powders and over-the-counter medications in glycerine is possible and described as steeping. This process is best conducted in a double boiler: one places the herbs and oil in a jar in a boiling pot rather than directly on the heat, as for tea infusion. Because the recovery yield and final concentrations of active substances are random, this practice remains experimental. 

Other illicit, highly addictive substances are also concerned by this vaping misuse. E-cigarettes can be used to consume other substances such as opium and crack cocaine [73]. Drugs diluted in glycerine have also been used with amphetamines such as ecstasy and methamphetamine, codeine, oxycodone (Percocet^®^), methylphenidate (Ritalin^®^), lysergic acid diethylamide (LSD), muscle relaxers, steroids or over-the-counter medications [49,74]. Alternatively, benzodiazepines such as alprazolam, phenazepam, midazolam and lorazepam are also commonly available in liquid form. To dilute the drug in liquid form, some illegal producers have used methamphetamine hydrochloride to convert the drug to a water-soluble methamphetamine hydrochloride powder that could later be dissolved in the polar solvents used in e-cigarette liquid refills such as glycerine and PG. Methamphetamine hydrochloride is slowly added to water and heated to barely below 100 °C to form a saturated solution. Although other recipes for crack cocaine dissolution in e-cigarette liquid refills have been reported, as with cannabis and heroin, dissolution causes flaws in solubility. The pH of the liquid refill is also important because the dissolved substance could turn back into unsmokable substances. These illegal drugs are more frequently consumed with inhalation pipes. The psychoactive effects obtained with these substances consumed by vaping have not been objectively and scientifically documented [75]. Only the personal and subjective feelings of experimentators have been reported. Problems with solubilisation and the random physicochemistry of illegal drugs in liquid refills are arguments against the vaping of illegal substances. However, if the efficiency of illegal drug vaping is improved, authorities and health policy stakeholders should pay attention to this new drug consumption, because more health problems will occur.

### 3.2. Illegal Drug Vaporising

Vaporisation comprises heating a solid/paste preparation containing an active substance at a moderate temperature, allowing the transfer of the active substance in a gaseous state but avoiding combustion/pyrolysis. Table cannabis vaporisers are currently commercially available (e.g., Volcano™, Storz & and Bickel GmbH) but are not very used for recreational illegal drug consumption. Recently, developments of pen-vaporisers based on pen-vaporisers available for nicotine inhalation and e-cigarettes have led to portable and lightweight vaporisers. Many of these portable vaporisers can be adapted to the consumption of cannabis extracts. E-cigarettes of different brands and of varied design may also be used or modified (MODs) for this purpose.

Several e-cigarettes equipped with special vaporisation chambers exist on the market: those able to atomise ground plant material, those made for cannabis wax vaporisation, and those intended for atomisation of liquid solutions i.e., cannavaping [7]. Only e-cigarettes able to atomise ground material are considered a legal method of use of these devices, such as therapeutic cannabis pen-vaporization. The two other types of modified e-cigarettes/vaporisers should not be considered for legal use because of the necessity of processing plant material (maceration, extraction, concentration, etc.). These treatments, even on medical cannabis, should not be authorised because they lead to concentrated forms of cannabinoids that cause health problems (high addiction risk, potential overdose, emetic effects). Recent cannabis consumption methods based on the butane extraction of cannabinoids, called “dabbing”, have been observed [76,77,78]. Solid-to-paste waxy cannabis concentrates of BHO, or “cannabis dabs”, can be vaporised with modified e-cigarettes/vaporisers [58]. However, every cannabis concentrate should undergo the step of decarboxylation of the inactive precursor THC-A into psychoactive THC. If this decarboxylation step is not preliminarily conducted, cannabis concentrates must be vaporised at 300–400 °C on a hot surface for decarboxylation before inhalation with a special pipe. Several modified e-devices can also perform this vaporisation although the majority are homemade devices. If the cannabis concentrate is previously decarboxylated, BHO/dabs are placed on coils or a titanium rod and heated by the e-device until vaporisation. Many e-vaporisers derived from the design of e-cigarettes are available in specialised shops and on the Internet. Based on these pen-vaporisers, other e-devices have been developed for recreational cannabis consumption such as e-hookahs and e-pipes. Such devices allow shared BHO consumption according to the number of pipes/tubes. However, these devices are a risk considering the high amount of active THC that can be delivered. These e-devices could also be used to vape illegal substances although their uses for this purpose have not been reported until now.

Based on these new cannabis consumption methods, other legal or illegal drugs can be consumed by vaporisation or dabbing. Theoretically, it is possible to produce plant concentrates of all legal and illegal plants such as those listed previously. Some Kratom (*Mitragyna speciose*; active substance: mitragynine) concentrates/dabs have been reported on the Internet with mixed psychoactive effects. Other users reported BHO attempts with ayahuasca (a mixture of *Banisteriopsis caapi*, *Psychotria viridis* and *Diplopterys cabrerana*; active substances: harmala alkaloids), datura (*Datura stramonium*; active substances: tropanic alkaloids including scopolamine, hyoscyamine and atropine), salvia (*Salvia divinorum*; active substance: salvinorine), iboga (*Tabernanthe iboga*; active substance: ibogaïne), khat (*Catha aedulis*; active substances: cathinone and cathine), psilocybe mushrooms (*Psilocybe* sp.; active substance: psilocybine), peyotl (*Lophophora williamsii*; active substances: mescaline, lophophorine), morning glory (*Ipomoea tricolor*; active substances: ergoline alkaloids) and lemon balm (*Melissa officinalis*; active substances: terpenic acids). These marginal products and effects are not well described on the Internet although consumption by vaporisation of these products is known [79]. Conversely, kava dabs (*Piper methysticum*; active substances: kavalactones) are marketed and available under names such as “Captain Kava”. This last product shows that other psychoactive plant dabs can be commercialised as well as homemade.

Finally, experiments with the vaporisation of crack (freebase cocaine), crystal meth/ice (methamphetamine hydrochloride) or freebase heroin (heroin hydrochloride) have also occurred with vaporisers. If dabs of some of these substances (cocaine and opium) are theoretically possible, such dabs have not been reported for the obvious reason of potential overdose. However, authorities should be aware of this possibility because volume reduction of drugs for transport is currently under investigation connected with illegal drug producers. The drugs should be less visible and more stable; however, cocaine or heroin dabs have not been widespread because the further dilution of the parent drug to “consumer” concentration remains difficult. Conversely, although dabs of amphetamines, cocaine or heroin are rarely described, vaporisation of these substances with glass pipes has been more widely documented. Amphetamines and particularly methamphetamine smoking/inhalation is a widespread mode of consumption although these methods may be far less rewarding because of the high temperature required to vaporise the drugs, which leads to a simultaneous inactivation of the active substance [80]. Indeed, despite these losses, this administration route offers the best bioavailability (90% for methamphetamines when smoked compared with 67% when ingested). Because substances such as amphetamines, crack cocaine and heroin freebase are smokable due to their relative low boiling point (from 90 °C for crack cocaine to 173 °C for heroin freebase, 200 °C for amphetamine and 215 °C for methamphetamine), these drugs could theoretically be vaporised in pen-vaporisers. Other cocaine and heroin freebases are more often smoked than amphetamines although these drugs also lead to losses. It is cheaper and safer to inhale these drugs rather than inject them intravenously. Therefore, these illegal drugs are easily vaporised and consumed by pipe inhalation without the need to control the heating temperature (lighter flame). However, the principle is quite similar to that of legal vaporisers with temperature control. 

Therefore, vaporisation of illegal crack, heroin or amphetamines is possible [81], has been tested and should provide higher psychoactive effects than inhalation because of the limitations of leaking due to the design of the vaporisation chamber. However, vaporisation of these drugs is not exceptionally popular because vaporisation is not more effective than cheap and handy inhalation pipes. For more discreet consumption, however, illegal drug vaporisation with e-vaporisers remains possible. Similarly, the use of pen-vaporisers to consume legal drugs for recreational purpose is also possible but not frequently reported. Glass pipes are commonly available for drug vaporisation, and the clean-up is easier. In fact, according to the excipient (sugars, etc.), drug vaporisation can cause e-device clogging, in which case, clean-up becomes tedious.

## 4. Therapeutic Drug Consumption with Electronic Devices

Although numerous experiments with illegal drug vaping and vaporising have been reported and are available on the Internet, few comments describe the benefits of e-devices for therapeutic outcomes, most likely because the innocuity of e-cigarettes and liquid refills remain unproven. Pharmaceutical and health policy stakeholders remain uninterested in these developments as long as clear sanitary positions are not taken and enacted by national laws and regulations. However, these decisions appear to be chimerical considering the speed with which this market is developing; the frequency of new designs, new technologies and new flavourings; and the possibility of modifying the current models.

Therapeutic cannabis vaporisation has been widely studied [82,83] because cannabis was deemed useful in the treatment of various pathologies [84]. Some studies have shown that the amount of THC in the vapour from a table vaporiser was similar to the amount of THC in the smoke of a cannabis cigarette, with a similar plasma THC concentration for both users [85,86,87]. Cannabis vaporisation is also advocated for patients who want to quit smoking or consuming drugs by significantly reducing the magnitude and frequency of their consumption and possibly decreasing withdrawal symptoms. Other works have also emphasised the less harmful pulmonary syndromes among vaporised cannabis users [88] and more efficient THC extraction from the plant [89]. However, if table cannabis vaporisers are easily available, their designs render these devices impractical because the vaporisers require plant material and suffer from a negative illegal drug image. Therefore, manufacturers attempted to solve this problem by developing portable pen-vaporisers. Consequently, it is not surprising that recent patents have been filed for vaporisers and pen-vaporisers for vaporising liquid for inhalation or to enhance the delivery of nicotine, THC, tobacco, cannabidiol or base alkaloid from an electronic cigarette or other vapour-producing devices [90,91,92]. In addition, cannabidiol-containing liquid refills have become legally available on the Internet. A shift from table vaporisers to pen-vaporisers can be observed; however, the literature concerning their therapeutic use remains scarce, and the frontier between pen-vaporiser and e-cigarette is unclear. While numerous studies concerning therapeutic cannabis vaporisation are available, only one study introducing the concept of therapeutic cannavaping has been reported [7]. Therefore, pen-vaporisers specifically designed for legal drug consumption are being developed although their therapeutic use remains marginal. Conversely, the misuse of electronic cigarettes to consume cannabis is widely described on the Internet. If table vaporisers have been miniaturised to pen-vaporisers to solve consumption problems (safety, reduced cost, discretion), the development of the misuse of e-cigarettes for recreational cannabis consumption can be explained by the same advantages. Consequently, if vaporisers are recommended for therapeutic cannabis consumption, cannavaping should also constitute a valuable medical alternative to cannabis smoking.

Legal vaporising and vaping, such as cannavaping, can offer new medical benefits [93,94]. Vaporising and vaping avoid smoking contaminants while maintaining the identical efficient bioavailability of inhalation route whereas ingested substances have random and sometimes poor bioavailability. In fact, ingested drugs first pass through the metabolism and become less active than inhaled compounds that have direct access to the bloodstream without being metabolised first. With regard to cannabis vaporisation, objective pharmacokinetic studies should be conducted to investigate the benefits of therapeutic vaping [95]. Indeed, therapeutic vaporisation should be able to deliver a more significant dose of active substance in a short time whereas therapeutic vaping should be used more for micro-delivery at various times during the day. No study is currently available concerning THC plasmatic concentration and pharmacokinetics among “cannavapers”. Recently, cannavapers have concluded that the electronic cigarette is a less efficient method of consuming cannabis than vaporisers [6]. The weaker amount of inhaled THC and the vaping protocol (shorter time) compared with the vaporising protocol may explain a less rewarding consumption although other studies should be conducted to evaluate the therapeutic outcome of electronic cigarettes. However, 45% of cannabis smokers and cannavapers have declared that cannavaping could help to reduce their consumption, and 37% reported no potential influence on their behaviour [6]. Cannavaping could therefore be useful for cannabis smoking cessation.

Similarly, based on cannavaping and vaping experiments with illegal drugs such as benzodiazepines, methaqualone, methylphenidate or opioids, legal drug vaporisation and vaping should be investigated more thoroughly by objective clinical studies. Avoiding the first-pass metabolism, the bioavailabilities of vaped drugs could be improved as well as the drug efficiency, particularly for drugs active at low concentration.

Nevertheless, taking drugs appears less negative with vaping and allows more discreet consumption of illegal drugs for recreational users. In addition to the huge benefits of pen-vaporisation and legal drug vaping, these new technologies have been, are and will again be misused by recreational users of illegal drugs. Therefore, certain problems should be anticipated. Because control of the e-device appears illusory, the only solution is to control the liquid refill composition. Solubilisation, homogeneity and physicochemical problems as well as random efficiency will undoubtedly occur in homemade products, and these flaws are deterrents for illegal drugs users. However, the manufacture of a legal liquid drug solution with a control on the composition (sufficiently diluted to avoid misuse but sufficiently concentrated to achieve therapeutic effects) could be optimised. Specific e-devices without open-access settings could be sold with the liquid refill of a legal drug.

Finally, therapeutic drug vaping sufficiently improved in order to provide safe and efficient drug administration requires study, and the problem of potential passive exposure by exhaled breath should be solved. Bioavailability investigations should be conducted to maximise the transfer of drugs from the mainstream aerosol to the organism, minimising the residual drug amount in exhaled breath. Secondary and even tertiary transfer (after redeposition of exhaled drugs on materials) should be controlled to assure that no (psycho)active amounts of drugs could be accidentally delivered to individuals in the neighbourhood of the vaper.

## 5. Conclusions

Although traces of contaminants can be detected in liquid refills and aerosols from e-cigarettes, the benefits of electronic cigarettes for changing the mentality of tobacco users are obvious. Some advocacy groups counsel that e-cigarettes should be recommended to smokers, and advertising should be specifically targeted to avoid the involvement of new vapers, particularly young people. However, e-devices such as pen-vaporisers, initially designed for therapeutic cannabis consumption, have been alternatively used to consume legal and illegal drugs for recreational purposes. Illegal drugs are available in various forms and can easily be consumed with the same advantages as e-cigarettes: safer, cheaper and more discreet. Solutions should be identified to avoid this phenomenon. If drug vaping is reported as less efficient than vaporising (glass pipe or pen-vaporiser) because of the lower amount of drugs and a lower magnitude of psychoactive effects, therapeutic outcomes may be identified for micro-dose treatments. The dangers of alternative use (for legal drugs) and misuse (for illegal drugs) are more serious with pen-vaporisers because the drugs are more concentrated. Therefore, vaping and vaporisation constitute new routes of administration for legal drugs that must be further investigated. Avoiding first-pass metabolism, the bioavailability of vaped and vaporised legal drugs should be higher, and the drug efficiency should be improved.
